# Risk variants in *BMP4 *promoters for nonsyndromic cleft lip/palate in a Chilean population

**DOI:** 10.1186/1471-2350-12-163

**Published:** 2011-12-19

**Authors:** José Suazo, Julio C Tapia, José Luis Santos, Víctor G Castro, Alicia Colombo, Rafael Blanco

**Affiliations:** 1 Department of Nutrition, Diabetes and Metabolism, Pontificia Universidad Católica de Chile, Santiago 8331150, Chile; 2 Program of Cellular and Molecular Biology, Institute of Biomedical Sciences, Faculty of Medicine, Universidad de Chile, Santiago 8700664, Chile; 3 Program of Human Genetics, Institute of Biomedical Sciences, Faculty of Medicine, Universidad de Chile, Santiago 8380453, Chile; 4 Program of Anatomy and Developmental Biology, Institute of Biomedical Sciences, Faculty of Medicine, Universidad de Chile, Santiago 8380453, Chile

## Abstract

**Background:**

Bone morphogenetic protein 4 gene (*BMP4*) plays a key role during maxillofacial development, since orofacial clefts are observed in animals when this gene is conditionally inactivated. We recently reported the existence of association between nonsyndromic cleft lip/palate (NSCLP) and *BMP4 *polymorphisms by detecting transmission deviations for haplotypes that include a region containing a *BMP4 *promoter in case-parent trios. The aim of the present study was to search for possible causal mutations within *BMP4 *promoters (BMP4.1 and BMP4.2).

**Methods:**

We analyzed the sequence of BMP4.1 and BMP4.2 in 167 Chilean NSCLP cases and 336 controls.

**Results:**

We detected three novel variants in BMP4.1 (c.-5514G > A, c.-5365C > T and c.-5049C > T) which could be considered as cleft risk factors due to their absence in controls. Additionally, rs2855530 G allele (BMP4.2) carriers showed an increased risk for NSCLP restricted to males (OR = 1.52; 95% C.I. = 1.07-2.15; p = 0.019). For this same SNP the dominant genotype model showed a higher frequency of G/G+G/C and a lower frequency of C/C in cases than controls in the total sample (p = 0.03) and in the male sample (p = 0.003). Bioinformatic prediction analysis showed that all the risk variants detected in this study could create new transcription factor binding motifs.

**Conclusions:**

The sex-dependent association between rs2855530 and NSCLP could indirectly be related to the differential gene expression observed between sexes in animal models. We concluded that risk variants detected herein could potentially alter *BMP4 *promoter activity in NSCLP. Further functional and developmental studies are necessary to support this hypothesis.

## Background

Nonsyndromic cleft lip with or without cleft palate (NSCLP [MIM 119530]) is one of the most common human craniofacial birth defects, with both genetic and environmental components involved in its etiology [1]. Its prevalence rate ranges from 1/300 to 1/2500 depending on the ethnic origin of the populations [2]. NSCLP presents a wide variety of primary and secondary medical complications in its rehabilitation. This fact plus medical costs and the emotional burden to patients and their families makes NSCLP a public health problem [3].

The identification of genetic risk factors in NSCLP has been the subject of intensive research in the last decades. In the past few years, the list of NSCLP candidate genes has rapidly increased and their study has been mainly focused in the search for coding mutations [4]. Some reports have estimated that the contribution of certain genes to NSCLP such as *MSX1 *accounts for 2%, *IRF6 *12-17% and 6% for the aggregate contribution of *FOXE1*, *GLI2*, *MSX2*, *SKI*, *SATB2 *and *SPRY2 *[5-8]. The increased knowledge that has emerged from all these reports has permitted an improvement in the knowledge of genetic factors involved in NSCLP, especially in families showing a familial aggregation of this disorder [9-11].

An attractive NSCLP candidate gene is bone morphogenetic protein 4 (*BMP4*, 14q22-23 in humans), a member of the transforming growth factor-beta superfamily. BMP signals regulate many aspects of skeletal development, including cartilage and bone formation during craniofacial and limb development [12]. Gong and Guo have reported that the *Bmp4 *expression localizes at the site of fusion of the mice facial prominences [13]. The conditional inactivation of *Bmp4 *in a transgenic mice line results in an isolated cleft lip [14]. The findings of these studies imply that the function of *Bmp4 *in the ectoderm of the facial processes is to regulate lip fusion.

In humans, few studies on the role of *BMP4 *in NSCLP have been reported. Lidral and Moreno performed a genome wide scan meta-analysis that showed evidence of linkage between NSCLP and the chromosomal region 14q21-24 [15]. Lin et al. performed an association study in a Chinese population using the non-synonymous single-nucleotide polymorphism (SNP) of *BMP4*, rs17563 (p.Val152Ala) and described that the C allele carriers showed an increased risk for NSCLP [16]. Recently, Jianyan et al. and Lin et al. have reported an interaction between rs17563 and environmental factors like maternal passive smoking in the expression of NSCLP [17,18]. The *BMP4 *coding sequence was analyzed by Suzuki et al. in a sample of patients with subepithelial, microform and overt cleft lip [19]. These authors detected missense and nonsense mutations in 0.7% of these patients which were absent in controls. All these findings support a role for genetic variation of *BMP4 *in the pathogenesis of NSCLP.

**Figure 1:**
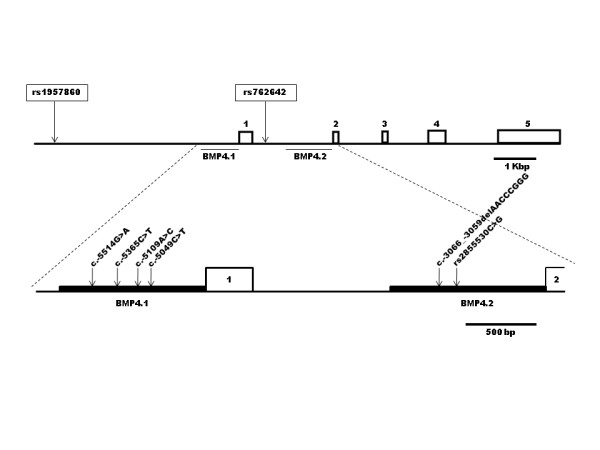
**Basic structure of human *BMP4 *gene and their two promoters**. Exons (boxes) are indicated by numbers 1 to 5. Upper panel shows the position of promoter 1 (BMP4.1) and promoter 2 (BMP4.2) and the location of the SNPs associated with NSCLP in a previous study (Suazo et al., 2010). Lower panel shows a more detailed view of the two promoters segment indicating the position of the novel and/or risk variants detected in the present study.

Our group recently reported a mutation screening analysis of *BMP4 *in a sample of 150 Chilean NSCLP case-parent trios. This analysis considered the coding regions (exons and exon-intron boundaries) and exclude regulatory regions. Due to the absence of causal mutations, we decided to genotype three SNPs (two intronic and one located 5 kb upstream to *BMP4*) in this same sample of triads. Significant deviations from expected transmissions were observed for haplotypes conformed by rs1957860 and rs762642 [20]. These polymorphisms delimitate a genomic region where a promoter and an enhancer of *BMP4 *are located [21]. Consequently, in this new study we searched for NSCLP risk variants within the two *BMP4 *promoters: BMP4.1 (located upstream from exon 1) and BMP4.2 (located upstream from exon 2) (Figure 1) [22] by direct sequencing in a case-control Chilean sample.

## Methods

### Patients and controls

Our study group consisted of 167 unrelated NSCLP cases and 336 controls. The distribution of cases by gender was 64% males and 36% females. Among cases, 115 were sporadic (9 cleft lip and 106 cleft lip and palate) and 52 had a positive family history (5 cleft lip and 47 cleft lip and palate). The patients were identified and interviewed during the course of clinical examinations between the years 2008 and 2011 in the following centers: Cleft Lip/Palate Clinic, School of Dentistry, University of Chile; Dental Service, Hospital Roberto del Rio; Cleft Lip/Palate Center, Hospital Exequiel Gonzalez Cortes; Maxillofacial Service, Hospital San Borja-Arriaran; Maxillofacial Service, Hospital Sotero del Rio (all of them located in the city of Santiago, Chile) and at the Corporation for the Help of Cleft Children (located in the city of La Serena, which is approximately 400 kilometers north from Santiago, Chile). In-depth interviews of at least three family members were conducted to provide detailed familiar information. A careful anamnesis was carried out to evaluate the use of teratogenic substances, such as phenytoin, warfarin and ethanol during pregnancy. The control group was recruited from blood donors of the Blood Bank, Hospital San Jose. After a careful interview those with a negative family history of orofacial clefts were incorporated in the study. The gender distribution of controls was 55% males and 45% females. The Institutional Review Boards of the Faculty of Medicine of the University of Chile and of the National Fund for Science and Technological Development (FONDECYT) approved our study and all participants gave their informed consent.

The contemporary urban Chilean population is mainly the result of the admixture between Amerindians (of Asian origin) and the Spanish settlers initiated in the XVI and XVII centuries [23]. The relationship between Amerindian admixture, socioeconomic strata, and prevalence of NSCLP has been extensively studied in Chile [24,25]. All individuals included in our study belong to the middle-low and low socioeconomic strata which show the highest rates of Amerindian admixture and NSCLP [25].

### Molecular Analysis

*Genomic DNA purification: *Genomic DNA was extracted from peripheral blood white cells according to the method described by Chomczynsky and Sacchi [26].

*BMP4 promoters sequencing: *the genomic segments corresponding to BMP4.1 and BMP4.2 were amplified by the polymerase chain reaction (PCR). The primers were designed using the on-line tool *Primer3 *http://frodo.wi.mit.edu/primer3/ taking as reference BMP4.1 and BMP4.2 sequences described by Van den Wijngaard et al. and deposited in GeneBank (accession numbers AF035427.1 and AF035428.1, respectively) [24]. BMP4.1 was described as a 1097 bp segment (chromosome position GRCh37:14:54424710-54423613) and BMP4.2 as a 1212 bp segment (chromosome position GRCh37:14:54422436-54421219) (Figure 1). Given that the maximum length for an appropriate sequence lecture of a segment is 850 bp, these promoters were amplified in two overlapping fragments. For this purpose AmpliTaq Gold^® ^360 (Applied Biosystems) was used as DNA polymerase applying 35 amplification cycles according to the manufacturer recommendations. Primers, length of amplified fragments and annealing temperature are listed in Additional File 1. All PCR products were visualized by 1.5% agarose gel electrophoresis and sent to Macrogen Inc. (Seoul, Korea) where they were sequenced using the forward primer. Samples with variants not previously described were also sequenced with the reverse primer to confirm these findings.

### Bioinformatic Analyses

The presence of SNPs previously described within BMP4.1 and BMP4.2 was examined using the *SNP BLAST *tool http://blast.ncbi.nlm.nih.gov/Blast.cgi?PROGRAM=blastn&BLAST_SPEC=SNP&BLAST_PROGRAMS=megaBlast&PAGE_TYPE=BlastSearch&SHOW_DEFAULTS=on&LINK_LOC=dbSNP_homepage. Sequencing results were analyzed by multiple alignments using the *ClustalW2 *program http://www.ebi.ac.uk/Tools/msa/clustalw2/ comparing them with the aforementioned reference sequences deposited in *GeneBank*. For sequence variants detected in cases but not in controls and for those associated with NSCLP, their capability to disrupt or create mammalian transcription factor binding motifs was predicted. For this purpose the softwares *Tfsitescan *http://www.ifti.org/cgi-bin/ifti/Tfsitescan.pl and *MatInspector *http://www.genomatix.de/en/index.html were used.

### Statistical Analyses

Allele and genotype frequencies of the BMP4.1 and BMP4.2 polymorphisms were estimated as simple proportions in cases and controls. An exact test to assess Hardy-Weinberg equilibrium implemented in the *Arlequin *statistical package was used in these polymorphisms [27]. To evaluate the association between NSCLP and BMP4.1 and/or BMP4.2 polymorphisms, allele and genotype Odds Ratio (OR) with 95% confidence intervals (C.I.) were estimated for the total sample and subdivided by gender. These analyses were performed using the *UNPHASED *program that applies a global likelihood-ratio significance test that does not require corrections for multiple comparisons [28]. Additionally, *UNPHASED *gives a likelihood-ratio test specific for each allele and genotype [28]. Parallel association analyses were carried out with *PLINK *software [29].

## Results

**Figure 2:**
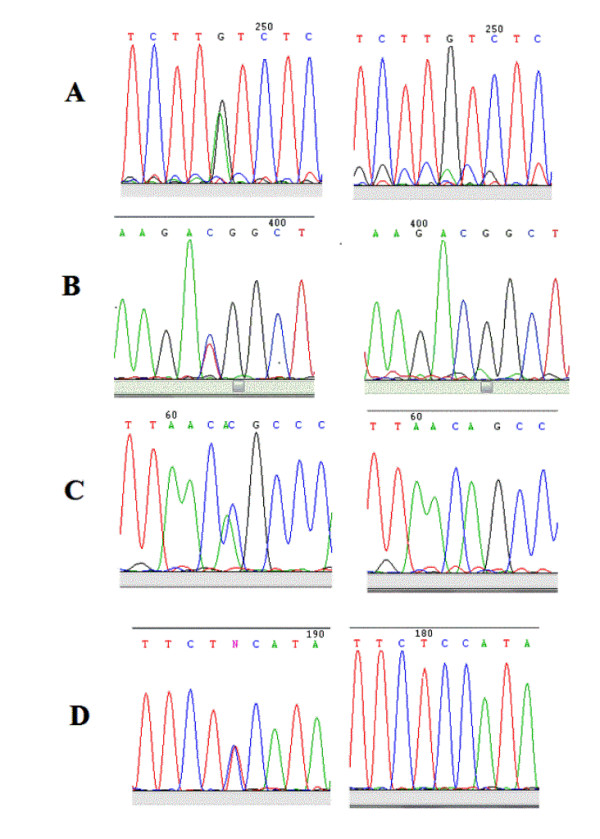
**Novel variants detected within BMP4**.1 promoter. A) c.-5514G > A. Left panel: sequence showing A/G change. Right panel: sequence without change. B) c.-5365C > T. Left panel: sequence showing T/C change. Right panel: sequence without change. C) c.-5109A > C. Left panel: sequence showing C/A change. Right panel: sequence without change. D) c.-5049C > T. Left panel: sequence showing T/C change. Right panel: sequence without change.

**Figure 3:**
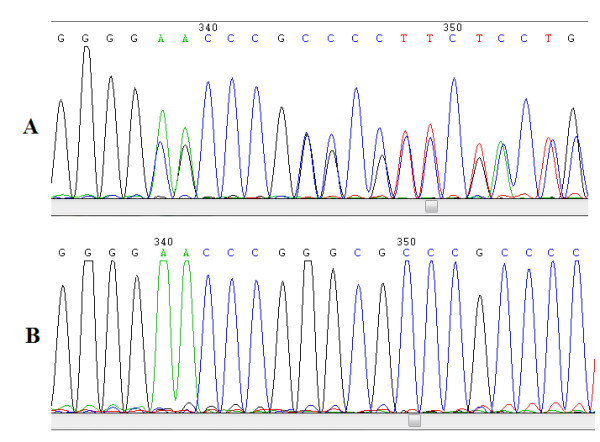
**A Novel variant detected within BMP4.2 promoter**. A) Sequence of a genomic region showing deletion AACCCGGG (c.-3066_c.-3059) within BMP4.2 promoter in a heterozygous individual. B) Sequence of the same genomic segment from an individual without deletion.

The analysis of both *BMP4 *promoters in cases detected the presence of novel genetic variants. Four of them were found in BMP4.1: c.-5514G > A, c.-5365C > T, c.-5109A > C and c.-5049C > T located at nucleotide 54424454, 54424305, 54424109 and 54423989 respectively (according to genome assembly GRCh37; Figures 1 and 2). The variants c.-5514G > A and c.-5109A > C were present in a heterozygous state in two different cases. The other two variants, c.-5365T and c.-5049T, were found in one male case which inherited them from his healthy mother possibly conforming a haplotype. Among these variants only c.-5109A > C was detected in one of the 336 controls (OR = 2.09; 95% C.I. = 0.13-33.52; p = 0.543) (data not shown). In BMP4.2 only one novel variant was detected corresponding to an eight base pairs deletion (c.-3066_-3059delAACCCGGG; Figures 1 and 3) in 3.9% of cases and in 5.9% of controls. No statistical differences between both group were observed (OR = 0.65; 95% C.I. = 0.33-1.27; p = 0.202; Table 1). Despite its frequency, this variant has not been previously described in genomic databases like *Ensembl *or *Human Mutation Database*.

**Table 1:** Polymorphic variants detected within BMP4.1 and BMP4.2 in NSCLP cases and controls

Promoter	Polymorphism	Genetic position	Chromosome position^a^	Variation	MAF^b^		Odds Ratio (95% C.I.)	p-value
					Cases	Controls		
BMP4.1	rs2855527	c.-5729	54424669	T > G	0.0032	0.0030	1.04 (0.09-11.55)	0.972
	rs77671695	c.-5098	54424038	T > G	0.040	0.055	0.72 (0.37-1.38)	0.322

	delAACCCGGG	c.-3066_c.-3059	54422006-54421999	ins > del	0.039	0.059	0.65 (0.33-1.27)	0.202
	rs2855530	c.-2977	54421917	C > G	0.418	0.369	1.23 (0.93-1.62)	0.146
BMP4.2	rs113141288	c.-2932	54421872	G > T	0.041	0.052	0.78 (0.41-1.50)	0.455
	rs76953585	c.-2679	54421619	C > T	0.044	0.054	0.79 (0.42-1.50)	0.484
	rs113562279	c.-2608	54421548	G > T	0.038	0.052	0.70 (0.35-1.37)	0.295

^a ^Chromosome position according to genome assembly GRCh37.

^b ^MAF: Minor allele frequency.

The polymorphic variants detected in BMP4.1 and BMP4.2 in NSCLP cases and controls in the total sample are shown in Table 2. Two SNPs were found in BMP4.1: rs2855527 and rs77671695, and four SNPs in BMP4.2: rs2855530, rs113141288, rs76953585 and rs113562279. No significant differences in the allele frequencies were observed between cases and controls (Table 1). When the sample was subdivided by gender, only the G allele of rs2855530 within BMP4.2 showed a greater frequency in male cases than in controls (OR = 1.52; 95% C.I. = 1.07-2.15; p = 0.019). This difference was not observed in female participants (OR = 0.78; 95% C.I. = 0.48-1.25; p = 0.297) (Table 2). (The just mentioned ORs and p-values had exactly the same values when were calculated by *UNPHASED *and *PLINK*).

**Table 2:** Allelic association between BMP4.1 and BMP4.2 polymorphisms and NSCLP subdivided by gender.

Promoter	Polymorphism	Males MAF^a ^		Odds Ratio (95% C.I.)	p-value	Females MAF^# ^		Odds Ratio (95% C.I.)	p-value
		**Cases**	**Controls**			**Cases**	**Controls**		

BMP4.1	rs2855527	0.0047	0.0027	1.74 (0.11-27.95)	0.692	0.000	0.0033	---	---
	rs77671695	0.042	0.057	0.73 (0.33-1.63)	0.444	0.035	0.052	0.65 (0.21-2.00)	0.454

	delAACCCGGG	0.029	0.060	0.47 (0.19-1.17)	0.099	0.059	0.058	1.01 (0.39-2.64)	0.982
	rs2855530	*0.456*	*0.356*	*1.52 (1.07-2.15)*	*0.019*	0.327	0.384	0.78 (0.48-1.25)	0.297
BMP4.2	rs113141288	0.043	0.051	0.84 (0.37-1.92)	0.685	0.036	0.054	0.66 (0.21-2.00)	0.456
	rs76953585	0.042	0.058	0.71 (0.32-1.59)	0.405	0.045	0.050	0.91 (0.32-2.57)	0.860
	rs113562279	0.037	0.058	0.63 (0.27-1.45)	0.273	0.037	0.046	0.78 (0.25-2.41)	0.295

^a ^MAF: Minor allele frequency.

Genotypes for BMP4.1 and BMP4.2 SNPs showed no significant deviations from Hardy-Weinberg expectations both in cases and controls (data not shown). The results of the genotype association analysis for BMP4.1 SNPs showed that only rs2855530 in BMP4.2 showed a positive association of C/G genotype (OR = 1.75; 95% C.I. = 1.14-2.69; p = 0.012) while an inverse relation was observed for the C/C genotype (OR = 0.79; 95% C.I. = 0.43-1.47; p = 0.020) (Table 3). *PLINK *software does not show an individual p-value for each genotype case-control comparison. However, the genotype association displayed a significant difference when G/G+C/G frequencies were compared with C/C frequencies. Thus, G/G+C/G were more frequent in cases than controls while C/C was more frequent in controls than cases (p = 0.032) (data not shown).

**Table 3:** Genotype association between BMP4.1 and BMP4.2 polymorphisms and NSCLP.

Promoter	Polymorphism	Genotypes	Frequency		Odds Ratio (95% C.I.)	p-value
			Cases	Controls		
	rs2855527	T/T	0.9938	0.9941	Ref.	
BMP4.1		T/G	0.0062	0.0059	1.04 (0.09-11.6)	0.972
		
	rs77671695	T/T	0.92	0.89	Ref.	
		T/G	0.08	0.11	0.71 (0.36-1.37)	0.308

	delAACCCGGG	Ins/Ins	0.92	0.88	Ref.	
		Ins/Del	0.08	0.12	0.63 (0.32-1.25)	0.189
		
		G/G	0.14	0.15	Ref.	
	rs2855530	C/G	0.56	0.44	*1.75 (1.14-2.69)*	*0.012*
		C/C	0.30	0.41	*0.79 (0.43-1.47)*	*0.020*
		
BMP4.2	rs113141288	G/G	0.92	0.90	Ref.	
		G/T	0.08	0.10	0.77 (0.39-1.50)	0.443
		
	rs76953585	C/C	0.92	0.89	Ref.	
		C/T	0.08	0.11	0.78 (0.41-1.51)	0.472
		
	rs113562279	G/G	0.92	0.89	Ref.	
		G/T	0.08	0.11	0.69 (0.34-1.36)	0.282

In male participants, the C/G genotype of rs2855530 showed an OR = 2.34 (95% C.I. = 1.33-4.09; p = 0.011) and the C/C genotype presented an OR = 0.51 (95% C.I. = 0.23-1.09; p = 0.003) while the same comparisons were non-significant for female participants (Table 4). Analysis performed by *PLINK *once again corroborate these results. Thus, the G/G+C/G versus C/C comparison was more significant between male cases and male controls than the total sample (p = 0.003).

**Table 4:** Genotype association between BMP4.1 and BMP4.2 polymorphisms and NSCLP subdivided by gender

			Males				Females			
**Promoter**	**Polymorphism**	**Genotypes**	**Frequency**		**Odds Ratio (95% C.I.)**	**p-value**	**Frequency**		**Odds Ratio (95% C.I.)**	**p-value**
			**Cases**	**Controls**			**Cases**	**Controls**		

	rs2855527	T/T	0.9906	0.9946	Ref.		1.0000	0.9934	Ref.	
BMP4.1		T/G	0.0094	0.0054	1.74 (0.10-28.16)	0.692	0.0000	0.0065		
		
	rs77671695	T/T	0.92	0.89	Ref.		0.93	0.89	Ref.	
		
		T/G	0.08	0.11	0.72 (0.32-1.63)	0.431	0.07	0.11	0.64 (0.21-2.01)	0.443

	delAACCCGGG	Ins/Ins	0.94	0.88	Ref.		0.88	0.88	Ref.	
		Ins/Del	0.06	0.12	0.45 (0.17-1.15)	0.090	0.12	0.12	1.01 (0.38-2.73)	0.982
		
		G/G	0.17	0.14	Ref.		0.08	0.16	Ref.	
	rs2855530	C/G	0.58	0.43	*2.34 (1.33-4.09)*	*0.011*	0.50	0.45	2.23 (0.71-7.08)	0.583
BMP4.2		C/C	0.25	0.43	*0.51 (0.23-1.09)*	*0.003*	0.42	0.39	2.21 (0.69-7.15)	0.655
		
	rs113141288	G/G	0.91	0.90	Ref.		0.93	0.89	Ref.	
		G/T	0.09	0.10	0.83 (0.36-1.93)	0.677	0.07	0.11	0.64 (0.20-2.01)	0.444
		
	rs76953585	C/C	0.92	0.88	Ref.		0.91	0.90	Ref.	
		C/T	0.08	0.12	0.69 (0.31-1.59)	0.392	0.09	0.10	0.91 (0.31-2.62)	0.857
		
	rs113562279	G/G	0.93	0.88	Ref.		0.93	0.91	Ref.	
		G/T	0.07	0.12	0.62 (0.26-1.44)	0.261	0.07	0.09	0.77 (0.24-2.44)	0.653

According to *Tfsitescan *and *MatInspector *softwares, the change observed in c.-5514 position generates a new site for GATA-1. The variant c.-5365C > T produces a sequence that can be potentially recognized by a RXR heterodimer transcription factor. The change T for C in c.-5049 introduced a previously inexistent site for TCF-1α. Finally, in the case of BMP4.2, this same bioinformatic analyses showed that rs2855530 G allele generated a Sp1 binding motif which is not detected when the C allele is present. Therefore, the bioinformatic analyses of the genetic variants c.-5514G > A, c.-5365C > T and c.-5049C > T within BMP4.1 and the SNP rs2855530 within BMP4.2 showed that they are capable of creating mammalian transcription factor binding motifs.

## Discussion

Regulatory variants are important in the understanding of the phenotypic diversity and the role they play in the susceptibility of complex diseases. However, it is noteworthy that these regulatory variants have not received the same scientific interest in comparison to coding variants [30]. According to the *Human Gene Mutation Database *http://www.hgmd.cf.ac.uk/ac/index.php approximately 1.6% of single base-pair substitutions described are regulatory mutations. Furthermore, there is abundant evidence indicating that regulatory SNPs (rSNPs) have an impact in the phenotypic diversity and can also affect disease susceptibility interacting with other variants located in their vicinity [31]. All these changes can potentially disrupt the DNA motifs recognized by transcription factors and consequently alter the normal processes of gene activation and/or transcriptional regulation [32]. In this context and taking into account the absent of NSCLP causal mutations in *BMP4 *coding regions plus the evidence of haplotype association reported by Suazo et al [20], the present report was focused in detecting NSCLP risk variants within both *BMP4 *promoters. In accordance with our previous report we found NSCLP risk variants within *BMP4 *promoters. Due to their frequencies it was impossible to establish if these novel variants are in linkage disequilibrium with those SNPs associated with NSCLP described by Suazo et al.

For BMP4.1 three novel substitutions were detected in cases (c.-5514G > A, c.-5365C > T and c.-5049C > T) which can be considered as potential susceptibility variants due to their absence in controls. Moreover, c.-5365T and c.-5049T were found in the same NSCLP case and they were inherited from his healthy mother. Therefore, although that this haplotype can be considered a risk factor it cannot produce the expression of NSCLP by itself and it would need other genetic variants and/or environmental factors absent in this case's mother. In tune with our findings, several mutations have been identified in NSCLP candidate genes mainly in sporadic cases [11]. For this reason these variants can be considered as private mutations from private families. The novel variants described in our study present the same characteristic shared by private mutations from private families.

In BMP4.2 we did not detect novel allelic variants. Regarding polymorphisms, SNP rs2855530 showed an association with NSCLP which presented a sexual dimorphism. Combining the results of association analyses using *UNPHASED *and *PLINK *softwares we can conclude that rs2855530 G allele and the G/G+C/G genotype (dominant model) should be considered risk factors but restricted to males due to their higher frequency in male cases than in male controls. On the other hand, the C/C genotype seems to represent a protective factor for male individuals given that its frequency is higher in male controls. Our group has previously reported a sexual or gender dimorphism for NSCLP where an STR allele of *MSX1 *gene showed significant differences between male cases and male controls [33]. Using animal models, sex-biased gene expression has been reported for gonadal and extragonadal tissues during embryogenesis where the major determinants of these differences are sex hormones [34,35]. The human adult face displays a sexual dimorphism which seems to be established in the first years of life but could depend on factors expressed in the prenatal life [36]. These evidences and our findings in the present study are closely linked with epidemiological findings showing a higher frequency of NSCLP in males than females.

The bioinformatic analysis of the risk variants predicts that they could create new transcription factor binding motifs which could be involved in NSCLP. The c.-5514A allele of BMP4.1 could introduce a new site for the hematopoietic transcription factor GATA-1 [37]. A different site for this factor has been reported within the human BMP4.1 promoter and it has been demonstrated that it produces a negative effect on *BMP4 *expression [22]. The c.-5365T allele also generates a consensus sequence for RXR transcription factor which is related with gene expression regulated by retinoic acid (RA). This situation may explain why teratogenic doses of RA induce cleft palate in *Rxr-α *knockout mice in a lower frequency than in wild-type animals [38]. The BMP4.1 c.-5049T allele introduces a novel site for TCF-1α, a canonical Wnt pathway effector which is expressed in the processes that originate the mice midface [39,40]. For BMP4.2, the bioinformatic analysis predicted that the rs2855530G allele generates a novel binding site for Sp1, an ubiquitous transcription factor. Sp1 can modulate the gene expression in cellular processes like differentiation, growth and apoptosis, among others [41]. Nevertheless, there is no information about Sp1 inactivation or overexpression related to craniofacial anomalies.

To our knowledge, the present study constitutes the first report detecting novel risk regulatory variants for a NSCLP candidate gene. In this context, three previous studies have associated rSNPs with this birth defect: this is the case of rs642961 located in an *IRF6 *enhancer, rs28999109 within the *PDGF-C *promoter, and rs16260 located in the *CDH1 *promoter [42-44]. The bioinformatic analysis for *IRF6 *and *PDGF-C *variants showed that the risk alleles disrupt potential transcription factor motifs. Nevertheless, reporter gene assays demonstrated that significant alterations in gene expression were detected only for the *PDGF-C *promoter variant [43]. Following the tendency set by these latter articles, functional studies are necessary to confirm our findings.

## Conclusions

In summary, we have detected three novel NSCLP potential causal variants in *BMP4 *promoters which could contribute to approximately 1.2% of this birth defect, as well as a risk SNP allele with a clear sex dependent association (rs2855530). These results are in concordance with our previous report showing the absence of potential causal mutations in the coding sequence of *BMP4*. The bioinformatic analyses have predicted that all these variants can potentially generate novel transcription factor recognition sites. In future reports will be necessary to confirm the *in vivo *capability of these variants to alter *BMP4 *expression using functional and developmental approaches.

## Competing interests

The authors declare that they have no competing interests.

## Authors' contributions

JS, JCT, JLS, AC and RB conceived and designed the study. JS and RB participated in the clinical interviews and sample collections JS and VGC developed protocols, conducted the laboratory experiments and bioinformatic analyses. JS and JLS performed the statistical analysis. JS, JCT, JLS, VGC, AC and RB drafted the manuscript. All authors read and approved the final manuscript.

## Pre-publication history

The pre-publication history for this paper can be accessed here:

http://www.biomedcentral.com/1471-2350/12/163/prepub

## Supplementary Material

Additional file 1 **Primer sequence and fragment sizes used for BMP4.1 and BMP4.2 PCR amplification and sequencing**. a table showing primers used for BMP4.1 and BMP4.2 PCR amplification and sequencing, fragment sizes and other PCR conditions. Click here for file

## References

[B1] Murray JC Schutte BC Cleft palate: players, pathways, and pursuits J Clin Invest 2004 113 1692 1700 15199400 10.1172/JCI22154 PMC420516

[B2] Vanderas AP Incidence of cleft lip, cleft palate, and cleft lip and palate among races: a review Cleft Palate J 1987 24 216 225 3308178

[B3] Lace B Vasiljeva I Dundure I Barkane B Akota I Krumina A Mutation analysis of the MSX1 gene exons and intron in patients with nonsyndromic cleft lip and palate Stomatologija 2006 8 21 24 16687911

[B4] Jugessur A Farlie PG Kilpatrick N The genetics of isolated orofacial clefts: from genotypes to subphenotypes Oral Dis 2009 15 437 453 10.1111/j.1601-0825.2009.01577.x 19583827

[B5] Jezewski PA Vieira AR Nishimura C Ludwig B Johnson M O'Brien SE Daack-Hirsch S Schultz RE Weber A Nepomucena B Romitti PA Christensen K Orioli IM Castilla EE Machida J Natsume N Murray JC Complete sequencing shows a role for MSX1 in non-syndromic cleft lip and palate J Med Genet 2003 40 399 407 10.1136/jmg.40.6.399 12807959 PMC1735501

[B6] Zucchero TM Cooper ME Maher BS Daack-Hirsch S Nepomuceno B Ribeiro L Caprau D Christensen K Suzuki Y Machida J Natsume N Yoshiura K Vieira AR Orioli IM Castilla EE Moreno L Arcos-Burgos M Lidral AC Field LL Liu YE Ray A Goldstein TH Schultz RE Shi M Johnson MK Kondo S Schutte BC Marazita ML Murray JC Interferon regulatory factor 6 (IRF6) gene variants and the risk of isolated cleft lip or palate N Engl J Med 2004 351 769 780 10.1056/NEJMoa032909 15317890

[B7] Srichomthong C Siriwan P Shotelersuk V Significant association between IRF6 820G→ A and nonsyndromic cleft lip with or without cleft palate in the Thai population J Med Genet 2005 42 e46 10.1136/jmg.2005.032235 15994871 PMC1736106

[B8] Vieira AR Avila JR Daack-Hirsch S Dragan E Félix TM Rahimov F Harrington J Schultz RR Watanabe Y Johnson M Fang J O'Brien SE Orioli IM Castilla EE Fitzpatrick DR Jiang R Marazita ML Murray JC Medical sequencing of candidate genes for nonsyndromic cleft lip and palate PLoS Genet 2005 1 e64 10.1371/journal.pgen.0010064 16327884 PMC1298935

[B9] Jugessur A Murray JC Orofacial clefting: recent insights into a complex trait Curr Opin Genet Dev 2005 15 270 278 10.1016/j.gde.2005.03.003 15917202 PMC2442458

[B10] Lidral AC Moreno LM Progress toward discerning the genetics of cleft lip Curr Opin Pediatr 2005 17 731 739 10.1097/01.mop.0000185138.65820.7f 16282779 PMC2752353

[B11] Vieira AR Unraveling human cleft lip and palate research J Dent Res 2008 87 119 125 10.1177/154405910808700202 18218836

[B12] Wan M Cao X BMP signaling in skeletal development Biochem Biophys Res Commun 2005 328 651 657 10.1016/j.bbrc.2004.11.067 15694398

[B13] Gong SG Guo C Bmp4 gene is expressed at the putative site of fusion in the midfacial region Differentiation 2003 71 228 236 10.1046/j.1432-0436.2003.710304.x 12694205

[B14] Liu W Sun X Braut A Mishina Y Behringer RR Mina M Martin JF Distinct functions for Bmp signaling in lip and palate fusion in mice Development 2005 132 1453 1461 10.1242/dev.01676 15716346

[B15] Marazita ML Murray JC Lidral AC Arcos-Burgos M Cooper ME Goldstein T Maher BS Daack-Hirsch S Schultz R Mansilla MA Field LL Liu YE Prescott N Malcolm S Winter R Ray A Moreno L Valencia C Neiswanger K Wyszynski DF Bailey-Wilson JE Albacha-Hejazi H Beaty TH McIntosh I Hetmanski JB Tunçbilek G Edwards M Harkin L Scott R Roddick LG Meta-analysis of 13 genome scans reveals multiple cleft lip/palate genes with novel loci on 9q21 and 2q32-35 Am J Hum Genet 2004 75 161 173 10.1086/422475 15185170 PMC1216052

[B16] Lin JY Chen YJ Huang YL Tang GP Zhang L Deng B Li M Ma H Luan RS Association of bone morphogenetic protein 4 gene polymorphisms with nonsyndromic cleft lip with or without cleft palate in Chinese children DNA Cell Biol 2008 27 601 605 10.1089/dna.2008.0777 18771417

[B17] Jianyan L Zeqiang G Yongjuan C Kaihong D Bing D Rongsheng L Analysis of interactions between genetic variants of BMP4 and environmental factors with nonsyndromic cleft lip with or without cleft palate susceptibility Int J Oral Maxillofac Surg 2010 39 50 56 10.1016/j.ijom.2009.10.010 19914800

[B18] Lin JY Luan RS Guo ZQ Lin XQ Tang HY Chen YP The correlation analysis between environmental factors, bone morphogenetic protein-4 and transforming growth factor beta-3 polymorphisms in nonsyndromic cleft lip with or without cleft palate Zhonghua Kou Qiang Yi Xue Za Zhi 2010 45 596 600 21176594

[B19] Suzuki S Marazita ML Cooper ME Miwa N Hing A Jugessur A Natsume N Shimozato K Ohbayashi N Suzuki Y Niimi T Minami K Yamamoto M Altannamar TJ Erkhembaatar T Furukawa H Daack-Hirsch S L'heureux J Brandon CA Weinberg SM Neiswanger K Deleyiannis FW de Salamanca JE Vieira AR Lidral AC Martin JF Murray JC Mutations in BMP4 are associated with subepithelial, microform, and overt cleft lip Am J Hum Genet 2009 84 406 411 10.1016/j.ajhg.2009.02.002 19249007 PMC2667991

[B20] Suazo J Santos JL Jara L Blanco R Association between bone morphogenetic protein 4 gene polymorphisms with nonsyndromic cleft lip with or without cleft palate in a Chilean population DNA Cell Biol 2010 29 59 64 10.1089/dna.2009.0944 19839778

[B21] Kawai S Sugiura T Characterization of human bone morphogenetic protein (BMP)-4 and -7 gene promoters: activation of BMP promoters by Gli, a sonic hedgehog mediator Bone 2001 29 54 61 10.1016/S8756-3282(01)00470-7 11472891

[B22] van den Wijngaard A van Kraay M van Zoelen EJ Olijve W Boersma CJ Genomic organization of the human bone morphogenetic protein-4 gene: molecular basis for multiple transcripts Biochem Biophys Res Commun 1996 219 789 794 10.1006/bbrc.1996.0312 8645259

[B23] Rothhammer F Lasserre E Blanco R Covarrubias E Dixon M Microevolution in human Chilean populations: Shovel-shape, mesial palatal version and other dental traits in Pewenche Indians Z Morphol Anthropol 1968 60 162 169 5681789

[B24] Valenzuela C On sociogenetic clines Ethol Sociobiol 1988 9 259 269 10.1016/0162-3095(88)90008-8

[B25] Palomino HM Palomino H Cauvi D Barton SA Chackraborty R Facial clefting and Amerindian admixture in populations of Santiago, Chile Am J Hum Biol 1997 9 225 232 10.1002/(SICI)1520-6300(1997)9:2<225::AID-AJHB9>3.0.CO;2-Z 28561520

[B26] Chomczynski P Sacchi N Single-step method of RNA isolation by acid guanidinium thiocyanate-phenol-chloroform extraction Anal Biochem 1987 162 156 159 2440339 10.1006/abio.1987.9999

[B27] Excoffier L Lischer HE Arlequin suite ver 3.5: a new series of programs to perform population genetics analyses under Linux and Windows Mol Ecol Resour 2010 10 564 567 10.1111/j.1755-0998.2010.02847.x 21565059

[B28] Dudbridge F Likelihood-based association analysis for nuclear families and unrelated subjects with missing genotype data Hum Hered 2008 66 87 98 10.1159/000119108 18382088 PMC2386559

[B29] Purcell S Neale B Todd-Brown K Thomas L Ferreira MA Bender D Maller J Sklar P de Bakker PI Daly MJ Sham PC PLINK: a tool set for whole-genome association and population-based linkage analyses Am J Hum Genet 2007 81 559 575 10.1086/519795 17701901 PMC1950838

[B30] Pastinen T Hudson TJ Cis-acting regulatory variation in the human genome Science 2004 306 647 650 10.1126/science.1101659 15499010

[B31] Chorley BN Wang X Campbell MR Pittman GS Noureddine MA Bell DA Discovery and verification of functional single nucleotide polymorphisms in regulatory genomic regions: current and developing technologies Mutat Res 2008 659 147 157 10.1016/j.mrrev.2008.05.001 18565787 PMC2676583

[B32] Cooper DN Human gene mutation in pathology and evolution J Inherit Metab Dis 2002 25 157 182 10.1023/A:1015621710660 12137225

[B33] Blanco R Chakraborty R Barton SA Carreño H Paredes M Jara L Palomino H Schull WJ Evidence of a sex-dependent association between the MSX1 locus and nonsyndromic cleft lip with or without cleft palate in the Chilean population Hum Biol 2001 73 81 89 10.1353/hub.2001.0002 11332647

[B34] Ellegren H Parsch J The evolution of sex-biased genes and sex-biased gene expression Nat Rev Genet 2007 8 689 698 10.1038/nrg2167 17680007

[B35] Bardin CW Catterall JF Testosterone: a major determinant of extragenital sexual dimorphism Science 1981 211 1285 1294 10.1126/science.7010603 7010603

[B36] Bulygina E Mitteroecker P Aiello L Ontogeny of facial dimorphism and patterns of individual development within one human population Am J Phys Anthropol 2006 131 432 443 10.1002/ajpa.20317 16596605

[B37] LaVoie HA The role of GATA in mammalian reproduction Exp Biol Med 2003 228 1282 1290 10.1177/153537020322801107 14681544

[B38] Nugent P Sucov HM Pisano MM Greene RM The role of RXR-alpha in retinoic acid-induced cleft palate as assessed with the RXR-alpha knockout mouse Int J Dev Biol 1999 43 567 570 10610030

[B39] Clevers H Wnt/beta-catenin signaling in development and disease Cell 2006 127 469 480 10.1016/j.cell.2006.10.018 17081971

[B40] Vendrell V Summerhurst K Sharpe J Davidson D Murphy P Gene expression analysis of canonical Wnt pathway transcriptional regulators during early morphogenesis of the facial region in the mouse embryo Gene Expr Patterns 2009 9 296 305 10.1016/j.gep.2009.03.001 19303461

[B41] Tan NY Khachigian LM Sp1 phosphorylation and its regulation of gene transcription Mol Cell Biol 2009 29 2483 2488 10.1128/MCB.01828-08 19273606 PMC2682032

[B42] Rahimov F Marazita ML Visel A Cooper ME Hitchler MJ Rubini M Domann FE Govil M Christensen K Bille C Melbye M Jugessur A Lie RT Wilcox AJ Fitzpatrick DR Green ED Mossey PA Little J Steegers-Theunissen RP Pennacchio LA Schutte BC Murray JC Disruption of an AP-2alpha binding site in an IRF6 enhancer is associated with cleft lip Nat Genet 2008 40 1341 1347 10.1038/ng.242 18836445 PMC2691688

[B43] Choi SJ Marazita ML Hart PS Sulima PP Field LL McHenry TG Govil M Cooper ME Letra A Menezes R Narayanan S Mansilla MA Granjeiro JM Vieira AR Lidral AC Murray JC Hart TC The PDGF-C regulatory region SNP rs28999109 decreases promoter transcriptional activity and is associated with CL/P Eur J Hum Genet 2009 17 774 784 10.1038/ejhg.2008.245 19092777 PMC2788748

[B44] Song Y Zhang S Association of CDH1 promoter polymorphism and the risk of non-syndromic orofacial clefts in a Chinese Han population Arch Oral Biol 2011 56 68 72 10.1016/j.archoralbio.2010.08.019 20880515

