# Supplementary material for: Risk variants in BMP4 promoters for nonsyndromic cleft lip/palate in a Chilean population
Source: BMC Med Genet. 2011 Dec 19;12:163. doi: 10.1186/1471-2350-12-163 (PMC3276445; doi:10.1186/1471-2350-12-163)
Supplement: Additional file 1 — Primer sequence and fragment sizes used for BMP4.1 and BMP4.2 PCR amplification and sequencing. a table showing primers used for BMP4.1 and BMP4.2 PCR amplification and sequencing, fragment sizes and other PCR conditions. [file 1471-2350-12-163-S1.DOC]

**Additional Table 1**

**Primer sequence and fragment sizes used for BMP4.1 and BMP4.2**

PCR amplification and sequencing

| **Fragment** | **Primer sequence (5`- 3`)** | **Fragment size** | **Annealing Temperature** |
| --- | --- | --- | --- |
| BMP4.1 Proximal | GCTCATTTACTGGGGTCTAC | 630 bp | 60ºC |
|  | TTCTTTCCTTCCTCCTCCTC |  |  |
| BMP4.1 Distal | TGGAGCAGAGGCTGTGATTT | 745 bp | 60ºC |
|  | ATGGAGAAAGGAGCTGTTGG |  |  |
| BMP4.2 Proximal | TTGCTCTTCCCAACCCTTTC | 787 bp | 60ºC |
|  | GATCTTAATGTGGCCGAGGT |  |  |
| BMP4.2 Distal | TCCAAGGATCGCGGTTTGTGAG | 734 bp | 64ºC |
|  | CTTCTTAGGGGCATTGGCAGGT |  |  |
